# Colorectal cancer surveillance in Hodgkin lymphoma survivors at increased risk of therapy-related colorectal cancer: study design

**DOI:** 10.1186/s12885-017-3089-8

**Published:** 2017-02-07

**Authors:** Lisanne S. Rigter, Manon C. W. Spaander, Leon M. Moons, Tanya M. Bisseling, Berthe M. P. Aleman, Jan Paul de Boer, Pieternella J. Lugtenburg, Cecile P. M. Janus, Eefke J. Petersen, Judith M. Roesink, John M. M. Raemaekers, Richard W. M. van der Maazen, Annemieke Cats, Eveline M. A. Bleiker, Petur Snaebjornsson, Beatriz Carvalho, Iris Lansdorp-Vogelaar, Katarzyna Jóźwiak, Hein te Riele, Gerrit A. Meijer, Flora E. van Leeuwen, Monique E. van Leerdam

**Affiliations:** 1 Department of Gastroenterology, Netherlands Cancer Institute, Plesmanlaan 121, Amsterdam, 1066 CX The Netherlands; 2 Department of Gastroenterology and Hepatology, Erasmus MC, University Medical Center, Rotterdam, The Netherlands; 3 Department of Gastroenterology and Hepatology, University Medical Center Utrecht, Utrecht, The Netherlands; 4 Department of Gastroenterology and Hepatology, Radboud University Medical Center, Nijmegen, The Netherlands; 5 Department of Radiation Oncology, Netherlands Cancer Institute, Amsterdam, The Netherlands; 6 Department of Hematology, Netherlands Cancer Institute, Amsterdam, The Netherlands; 7 Department of Hematology, Erasmus MC, University Medical Center, Rotterdam, The Netherlands; 8 Department of Radiation Oncology, Erasmus MC Cancer Institute, Rotterdam, The Netherlands; 9 Department of Hematology, University Medical Center Utrecht, Utrecht, The Netherlands; 10 Department of Radiation Oncology, University Medical Center Utrecht, Utrecht, The Netherlands; 11 Department of Hematology, Radboud University Medical Center, Nijmegen, The Netherlands; 12 Department of Radiation Oncology, Radboud University Medical Center, Nijmegen, The Netherlands; 13 Division of Psychosocial Research and Epidemiology, Netherlands Cancer Institute, Amsterdam, The Netherlands; 14 Department of Pathology, Netherlands Cancer Institute, Amsterdam, The Netherlands; 15 Department of Public Health, Erasmus MC, University Medical Center, Rotterdam, The Netherlands; 16 Department of Epidemiology, Netherlands Cancer Institute, Amsterdam, The Netherlands; 17 Division of Biological Stress Response, Netherlands Cancer Institute, Amsterdam, The Netherlands

**Keywords:** Hodgkin lymphoma, Surveillance, Colonoscopy, Colorectal neoplasia, Stool (DNA) test, Carcinogenesis

## Abstract

**Background:**

Second primary malignancies are a major cause of excess morbidity and mortality in cancer survivors. Hodgkin lymphoma survivors who were treated with infradiaphragmatic radiotherapy and/or high-dose procarbazine have an increased risk to develop colorectal cancer.

Colonoscopy surveillance plays an important role in colorectal cancer prevention by removal of the precursor lesions (adenomas) and early detection of cancer, resulting in improved survival rates. Therefore, Hodgkin lymphoma survivors treated with infradiaphragmatic radiotherapy and/or high-dose procarbazine could benefit from colonoscopy, or other surveillance modalities, which are expected to reduce colorectal cancer incidence and mortality. Current knowledge on clinicopathological and molecular characteristics of therapy-related colorectal cancer is limited. The pathogenesis of such colorectal cancers might be different from the pathogenesis in the general population and therefore these patients might require a different clinical approach.

We designed a study with the primary aim to assess the diagnostic yield of a first surveillance colonoscopy among Hodgkin lymphoma survivors at increased risk of colorectal cancer and to compare these results with different screening modalities in the general population. Secondary aims include assessment of the test characteristics of stool tests and evaluation of burden, acceptance and satisfaction of CRC surveillance through two questionnaires.

**Methods/Design:**

This prospective multicenter cohort study will include Hodgkin lymphoma survivors who survived ≥8 years after treatment with infradiaphragmatic radiotherapy and/or procarbazine (planned inclusion of 259 participants). Study procedures will consist of a surveillance colonoscopy with removal of precursor lesions (adenomas) and 6–8 normal colonic tissue biopsies, a fecal immunochemical test and a stool DNA test. All neoplastic lesions encountered will be classified using relevant histomorphological, immunohistochemical and molecular analyses in order to obtain more insight into colorectal carcinogenesis in Hodgkin lymphoma survivors. The Miscan-model will be used for cost-effectiveness analyses.

**Discussion:**

Evaluation of the diagnostic performance, patient acceptance and burden of colorectal cancer surveillance is necessary for future implementation of an individualized colorectal cancer surveillance program for Hodgkin lymphoma survivors. In addition, more insight into treatment-induced colorectal carcinogenesis will provide the first step towards prevention and personalized treatment. This information may be extrapolated to other groups of cancer survivors.

**Trial registration:**

Registered at the Dutch Trial Registry (NTR): NTR4961.

## Background

### Colorectal cancer risk in cancer survivors

Because of the improvements of cancer treatment over recent decades, cancer survival has greatly increased. A major and serious complication of cancer treatment is the occurrence of second primary malignancies, which accounts for approximately 18% of all cancers in the Western world [1, 2]. The development of second primary malignancies is influenced by multiple factors, including genetic predisposition, environmental factors and lifestyle factors. Moreover, certain anticancer treatments have the (paradoxical) capability to cause cancer, due to mutagenic and genome destabilizing effects.

Hodgkin lymphoma (HL) survivors have a substantial excess risk of developing colorectal cancer (CRC) [3–8]. The risk of CRC is also increased in survivors of many other malignancies, including survivors of testicular cancer, Wilms tumor, central nervous system malignancies and bone cancer [6–9]. The excess risk of CRC is strongly related to primary cancer treatment with radiotherapy or alkylating agents [5, 7, 8, 10]. A recent analysis of a Dutch multicenter HL cohort showed an increased incidence of CRC in long-term survivors of HL compared with the general population (SIR 2.4 (95% CI 1.8–3.2). HL survivors who were treated with high dose (>4.2 g/m2) procarbazine, or with infradiaphragmatic radiotherapy in combination with chemotherapy have an even higher risk (SIR 4.3 (95% CI 2.9–6.1) and 5.7 (95% CI 3.7–8.2), respectively) [3, 5].

### Colorectal cancer surveillance

HL survivors have a clearly increased risk of developing CRC for a prolonged period, starting 10 years up to over 30 years after receiving infradiaphragmatic radiotherapy and/or procarbazine [5]. These HL survivors may therefore benefit from a CRC surveillance program. Colonoscopy surveillance is important for CRC prevention in high-risk populations by early detection and removal of the precursor lesions (adenomas) with malignant potential, resulting in improved survival rates [11–13]. Surveillance programs in high-risk groups are different from screening programs in the general population, for which the fecal occult blood test-based program is the method of choice in the European Union [14]. However, the fecal occult blood test is inadequate for high-risk populations because of the relatively low sensitivity for advanced adenomas.

In the Netherlands, surveillance is performed in specific high-risk populations, like those with familial CRC or Lynch syndrome [15]. In current Dutch clinical practice, no colonoscopy surveillance program exists for HL survivors at high risk of CRC, because important requirements for screening/surveillance are not met, i.e. the presence of a recognizable latent stage of disease and knowledge of natural history. The pattern of development of a precursor lesion into cancer has substantial influence on the preventive effects of colonoscopy surveillance, and this pattern is unknown for CRCs that develop in HL survivors. Flat adenomas in the presence of chronic radiation colitis have been described in case reports on cancer survivors [16, 17]. In addition, a recent review reported a high frequency of mucinous adenocarcinomas in case reports on radiotherapy-associated rectal cancer, suggesting a difference in tumor biology as compared with sporadic CRCs [18].

It is thus important to personalize CRC prevention programs for different subgroups.

Because of the high risk of CRC in HL survivors, and the lack of data on the pathogenesis of these cancers, we designed a prospective cohort study that evaluates diagnostic yield and test characteristics of colonoscopy surveillance in HL survivors. These characteristics will also be evaluated for stool tests. In addition, the cost-effectiveness and patient perception of CRC surveillance will be evaluated. This knowledge could lead to a personalized CRC surveillance program for HL survivors.

## Methods

### Objectives

The objective of this study is to provide information on the potential benefit of a personalized CRC surveillance program for HL survivors at increased risk of developing CRC.

The primary aim is to assess the diagnostic yield of advanced colorectal neoplasia detected by colonoscopy among HL survivors at increased risk of CRC. Advanced colorectal neoplasia is defined as an adenoma with high grade dysplasia, ≥25% villous component or ≥10 mm diameter or CRC.

Secondary aims are to assess the test characteristics of stool tests (fecal immunochemical test (FIT), and stool DNA (sDNA test)) and to examine patient perception (burden, acceptance and satisfaction) and cost-effectiveness of CRC surveillance in HL survivors. In addition, the clinicopathological and molecular characteristics of therapy-related colorectal neoplasia will be evaluated.

The Medical Ethics Committee of the Netherlands Cancer Institute approved this study protocol and the study is currently ongoing. We adhered to SPIRIT guidelines.

### Study design

#### Population

This prospective multicenter cohort study will include patients from a large cohort of HL survivors who were treated for HL after 1965. HL treatment data and follow-up data were collected as previously described [3, 5, 19, 20].

Patients who meet the inclusion criteria will be invited for study participation in one of the Dutch study centers (The Netherlands Cancer Institute, Amsterdam, Erasmus MC, University Medical Center, Rotterdam, University Medical Center Utrecht, Utrecht and Radboud University Medical Center, Nijmegen). The inclusion criteria consist of the treatment of primary or recurrent HL consisting of at least one of the following treatments:

1. infradiaphragmatic radiotherapy consisting of at least para-aortic and iliac fields
2. chemotherapy containing a cumulative procarbazine dose of ≥2.8 g/m^2^
3. infradiaphragmatic radiotherapy (any field (s)) and chemotherapy (any regimen)

Additional inclusion criteria are HL diagnosis at the age of 16–50 years, survival of at least 8 years after this treatment, current age of 25 years or older and life expectancy of 5 years or more. Patients who meet one of the following criteria will be excluded: proctocolectomy, colonoscopy surveillance for other indications (including hereditary CRC syndrome, familial CRC syndrome, inflammatory bowel disease, colorectal adenoma, history of CRC), colonoscopy in the past 5 years, on-going cytotoxic treatment or radiotherapy for malignant disease, coagulopathy (prothrombin time > 50% of control; partial thromboplastin time > 50 s) or anticoagulants (vitamin K antagonists or new oral anticoagulants) that cannot be stopped, comorbidity leading to an impaired physical performance (World Health Organization performance status 3–4) or mental retardation and no informed consent.

The population of HL survivors in the four participating centers consists of approximately 850 eligible participants. Patients will be invited by their treating physician, either a radiation oncologist or a medical oncologist/hemato-oncologist. When patients are no longer under surveillance, they will be invited to a lymphoma Survivorship Care Clinic to inform them about the risk of late effects of Hodgkin lymphoma treatment and to perform surveillance according to standardized follow-up guidelines [21]. These Survivorship Care Clinics are being set up by a national consortium i.e. the BETER consortium (Better care after HL, Evaluation of long-term Treatment Effects and screening Recommendations). This consortium aims to reduce morbidity and mortality of late adverse effects of HL treatment by a survivorship care program in order to improve life expectancy and quality of life of HL survivors. At the Survivorship Care Clinic, eligible participants will be invited for participation in this CRC surveillance research project.

#### Control population

For the diagnostic yield of colonoscopy, FIT and sDNA test, comparison with the asymptomatic Dutch general population will be performed using the data of the COCOS study [22]. In this study primary colonoscopy screening was performed in 1276 asymptomatic individuals out of 5924 randomly selected invitees between 50 and 75 years of age. Advanced neoplasia (defined as an adenoma with high grade dysplasia, ≥25% villous component or ≥10 mm diameter or CRC) was detected in 9% of individuals in this population. 1256/1276 individuals also performed a FIT and 1060/1276 collected a feces sample for a sDNA test [23].

For the molecular profile, data will be compared with the Dutch general population using existing data of sporadic CRC under the age of 70 years [24].

#### Sample size calculation

The main endpoint of the study is the diagnostic yield of advanced colorectal neoplasia among HL survivors detected by surveillance colonoscopy, which will be compared with the diagnostic yield performed in the general population [22].

Based on a 9% prevalence of advanced colorectal neoplasia in the asymptomatic general population, an increase to 15% of more in HL survivors after adjustment for age would be a significant change. To detect such a difference with 80% power, we need to include at least 259 study participants (based on the two-sided test for two independent proportions with 5% significance level). An interim analysis will be performed after the inclusion of 100 participants. At this point, when the detected prevalence of advanced colorectal neoplasia in the study population is 30% or higher, the difference between the two groups will be considered significant with a power of 80% and alpha level of 0.0006 (based on the group-sequential tests for two proportions).

#### Cost-effectiveness analyses

We will use the well-established microsimulation screening analysis (MISCAN) model to determine the cost-effectiveness of colonoscopy, FIT and sDNA test surveillance in HL survivors compared to FIT screening, e.g. the Dutch CRC screening program [25–28].

This MISCAN model will be performed to estimate the size of health benefits and costs of a surveillance program. In this mathematical model, a large number of fictitious individual life histories are simulated in each of which several colorectal lesions can emerge. The influence of the implementation of a surveillance program will be simulated, which can change some of the life histories.

This model will be able to estimate incidence, prevalence and mortality of CRC, and the results and effects of surveillance. The costs and the number of life-years gained for the population with and without the implementation of surveillance will be calculated.

The MISCAN model will be used to estimate the optimal time of initiation, interval and frequency of colonoscopies. These results will be used for the CRC surveillance recommendation in HL survivors.

### Study procedures

#### Colonoscopy

All patients will receive oral and standard written information about the preparation and the colonoscopy by their treating physician or by the gastroenterologist. The preparation will be performed as for routine colonoscopy by ingesting a commonly prescribed oral electrolyte lavage solution. Standard methods of conscious sedation (midazolam and/or fentanyl citrate or propofol) and cardiopulmonary monitoring will be used during the procedure. The colonoscopy will be performed by experienced gastroenterologists (>1000 colonoscopies and certified for performing colonoscopies in the Dutch population-based CRC screening program). Quality measures of colonoscopy will include registration of location of deepest insertion, withdrawal time, quality of preparation (Boston scale), colonoscopy difficulty (5-point scale), patient discomfort (Gloucester comfort scale) and level of sedation (Leeds score).

In the presence of colorectal neoplasia, polypectomy will be performed or biopsies will be taken according to standard protocol. The following data will be collected concerning colorectal neoplasia: number, morphology (Paris classification), size (measured with an open biopsy forceps), location (cecum, ascending, hepatic flexure, transverse, splenic flexure descending, sigmoid or rectum) and details about the polypectomy.

In the presence of a polyp of ≥10 mm, field biopsies will be taken and a fragment of the polyp will be allocated for study purposes after routine pathological evaluation. Four biopsies of normal mucosa of the transverse colon and two to four biopsies of normal mucosa of the descending colon will be taken (in both patients who did and who did not receive infradiaphragmatic irradiation). The transverse colon is the colonic segment that usually receives the highest dose of irradiation in infradiaphragmatic radiotherapy, whereas the descending colon receives a low dose of irradiation. In case of an additional surgical resection of a neoplastic lesion, part of the resection specimen will be allocated for study purposes. Study material will be stored at the participating hospitals and shipped to the Netherlands Cancer Institute for analyses.

#### Histology, immunohistochemistry and molecular pathology

Routine histological evaluation of all colorectal neoplasia will be performed by experienced gastro-intestinal pathologists. Immunohistochemical and molecular analyses will be performed on advanced neoplastic lesions. The advanced colorectal neoplasia will be immunohistochemically stained for the presence of mismatch repair proteins (MLH1, MSH2, MSH6 and PMS2). DNA will be isolated and microsatellite instability analysis will be performed using a standard Pentaplex PCR kit. A multiplex ligation-dependent probe amplification kit will be used to assess the CpG island methylator phenotype, which detects the methylation status of promoter regions of 8 different genes (*CACNA1G, CDKN2A, CRABP1, IGF2, MLH1, NEUROG1, RUNX3 and SOCS1*). Mutation analysis of *KRAS* and *BRAF* will be performed by using Sequenome MassArray and/or high-resolution melting and sequencing. Further analyses of the advanced neoplastic lesions will depend on the results of an ongoing study on a retrospective cohort of CRCs in HL survivors.

#### Biopsies

The characteristics of the colonic mucosa biopsies in both the transverse and descending colon will be analyzed and stratified by treatment to evaluate the effect of irradiation on endoscopic normal mucosa. These analyses will depend on the results of abovementioned analyses, and will include the evaluation of mismatch repair function.

#### Questionnaires

The participant will be sent the first questionnaire prior to colonoscopy. This questionnaire will evaluate risk factors for CRC other than prior cancer treatment, the physical and mental functioning of the patient (EQ-5D, cancer worry scale, hospital anxiety and depression scale) and the expected burden of colonoscopy.

The second questionnaire will be sent to the participant a week after the colonoscopy. This questionnaire will include questions about the physical and mental functioning of the patient (EQ-5D, cancer worry scale, hospital anxiety and depression scale) and the experienced burden of colonoscopy. The questionnaires on the expected burden and experienced burden will consist of information on the mental and physical burden, including embarrassment and pain during both the bowel preparation and the colonoscopy procedure. The burden of colonoscopy will be compared with the burden of a colonoscopy in the general population [29].

#### Stool tests

A whole stool sample will be collected and a FIT will be performed on the sample. The whole-stool samples will be collected in a dedicated collection kit (Exact Sciences corporation, Madison, USA), which includes a buffer to stabilize and preserve the sample that will be taken prior to the start of bowel preparation for the surveillance colonoscopy. All kits will need to be at the laboratory for analysis within 72 h after sample production. Samples that are received after this time interval will be excluded from the study. Samples will be homogenized, aliquoted and stored at −80 °C.

The stool tests will include a FIT and a sDNA test for the detection of methylation and mutation markers. The sensitivity, specificity, positive predictive value and negative predictive value will be calculated using the colonoscopy as a reference standard. Also, results will be compared to the detection rates in the asymptomatic general population [23].

## Discussion

This protocol describes a multicenter prospective cohort study that assesses the value of colonoscopy surveillance and the additional value of FIT and sDNA tests in a high-risk population of HL survivors. For the development of a personalized surveillance program, we need to evaluate the diagnostic yield, test characteristics, patient perception and cost-effectiveness of various methods (i.e. colonoscopy, FIT, sDNA). The study is currently ongoing and is expected to complete inclusion of participants at the end of 2017.

Colonoscopy screening programs are implemented in the general population in many countries, and intensified personalized surveillance programs exist for various high-risk groups.

The pattern of development of a precursor lesion into cancer has substantial influence on the preventive effects of colonoscopy surveillance. The most common pathway of the development of CRC is through a protrude polypoid neoplastic lesion that can easily be detected and resected endoscopically. Nevertheless, a subset of precursor lesions is easily missed and/or difficult to resect, e.g. sessile or flat lesions, or dysplastic foci like those that occur in inflammatory bowel disease [30]. Also, microsatellite instable tumors are likely to develop through an accelerated growth pattern that emphasizes the need for a higher frequency of surveillance [31].

Because of these differences in development of a precursor lesion into cancer and the differences in CRCs risks, each high-risk group surveillance recommendation includes specific characteristics (e.g. starting age, frequency and technique).

Most cancer survivors at increased risk of therapy-related CRC are not aware of their increased CRC risk and do not receive CRC surveillance. The American Children’s Oncology Group (COG) recommends colonoscopy surveillance in survivors of childhood cancer who received at least 30 Gray of abdominal radiotherapy, starting 10 years after radiotherapy or at the age of 35 (whichever occurs last) and repeating every 5 years [24]. The Dutch lymphoma survivor consortium and childhood cancer survivor consortium, however, did not include CRC surveillance in their guidelines because of the absence of existing data for this specific category of patients. Evidence is lacking for CRC risk stratification of HL survivors, and it is not known if the pathogenesis of radiation- and chemotherapy-induced CRCs is similar to the pathogenesis of CRC in the general population [18, 32]. In addition, the COG recommendation is limited to childhood cancer survivors and it does not include survivors who have been treated with chemotherapy, but only with abdominal radiotherapy [3, 7, 10, 33]. Also, the effectiveness of surveillance according to this recommendation is not clear, and Daniel et al. reported that the adherence to this recommendation was less than 30% [34].

HL survivors have an increased risk of developing CRC for a prolonged period in their lives, [5] which makes CRC surveillance in this population likely beneficial. Not all studies show comparable increased CRC risks in survivors of various cancer types, including HL. However, these studies frequently lacked follow-up time (<10 years) and/or stratification for treatment differences, e.g. treatment regimen and dosage. This may have led to an underestimation of the risk of CRC in cancer survivors who were treated with infradiaphragmatic irradiation and/or alkylating agents [5, 7, 8, 10].

There is little information on the clinical characteristics of therapy-related CRC. Youn et al. compared the survival of first primary CRC patients with 70 stage II-IV colon cancer patients who were treated for HL. After adjusting for stage, overall survival and CRC-specific survival were reduced in the small population of HL survivors (HR 1.46 (95% CI 1.1–2.0) and HR 1.37 (95% CI 0.96–1.96), respectively) [8]. In addition to the excess risk of morbidity and mortality caused by CRC, HL survivors have an increased risk of cardiovascular disease and other second malignant neoplasms [4, 20]. The population that will benefit from CRC surveillance may therefore be smaller than expected because of these competing risks.

In addition to the insight into the therapy-related colorectal carcinogenesis that will be obtained in the present study, we are currently performing a retrospective study on the histopathological and molecular characteristics of therapy-related CRC already diagnosed in the HL survivor cohort [3, 5]. The results of this study will influence the analyses on the advanced colorectal neoplasia detected in this prospective study.

In conclusion, this protocol describes a prospective cohort study that evaluates diagnostic yield of advanced colorectal neoplasia, the most optimal surveillance method, patient acceptance and cost-effectiveness of CRC surveillance in HL survivors. The results will provide necessary information for the development of a personalized CRC surveillance program in this high-risk population. This information may be extrapolated to other groups of cancer survivors at increased risk of CRC, such as childhood cancer survivors [8, 10]. The incidence of advanced colorectal neoplasia over time cannot be evaluated, because this CRC surveillance study is limited to a first colonoscopy. Therefore, we intend to perform a follow-up study to evaluate a second surveillance round.

## Abbreviations

CI: Confidence interval
COG: Children’s Oncology Group
CRC: Colorectal cancer
FIT: Fecal immunochemical test
FOBT: Fecal occult blood test
HL: Hodgkin lymphoma
HR: Hazard ratio
MISCAN: Microsimulation screening analysis
PY: Person years
sDNA: Stool DNA
SIR: Standardized incidence ratio

## References

[1] National Cancer Institute SEER Cancer Statistics Review, 1975–2007 2010. Available from: http://seer.cancer.gov/csr/1975_2007/

[2] Morton LM, Swerdlow AJ, Schaapveld M, Ramadan S, Hodgson DC, Radford J (2014). Current knowledge and future research directions in treatment-related second primary malignancies. EJC Suppl.

[3] van AM E, Schaapveld M, Janus CP (2013). Long-term risk of colorectal cancer in patients treated for Hodgkin’s lymphoma. Gastroenterology Abstract.

[4] Hodgson DC, Gilbert ES, Dores GM, Schonfeld SJ, Lynch CF, Storm H (2007). Long-term solid cancer risk among 5-year survivors of Hodgkin’s lymphoma. J Clin Oncol.

[5] Schaapveld M, Aleman BM, van Eggermond AM, Janus CP, Krol AD, van der Maazen RW (2015). Second cancer risk Up to 40 years after treatment for Hodgkin’s lymphoma. N Engl J Med.

[6] Reulen RC, Frobisher C, Winter DL, Kelly J, Lancashire ER, Stiller CA (2011). Long-term risks of subsequent primary neoplasms among survivors of childhood cancer. JAMA.

[7] Henderson TO, Oeffinger KC, Whitton J, Leisenring W, Neglia J, Meadows A (2012). Secondary gastrointestinal cancer in childhood cancer survivors: a cohort study. Ann Intern Med.

[8] Nottage K, McFarlane J, Krasin MJ, Li C, Srivastava D, Robison LL (2012). Secondary colorectal carcinoma after childhood cancer. J Clin Oncol.

[9] Travis LB, Fossa SD, Schonfeld SJ, McMaster ML, Lynch CF, Storm H (2005). Second cancers among 40,576 testicular cancer patients: focus on long-term survivors. J Natl Cancer Inst.

[10] Tukenova M, Diallo I, Anderson H, Hawkins M, Garwicz S, Sankila R (2012). Second malignant neoplasms in digestive organs after childhood cancer: a cohort-nested case–control study. Int J Radiat Oncol Biol Phys.

[11] Nishihara R, Wu K, Lochhead P, Morikawa T, Liao X, Qian ZR (2013). Long-term colorectal-cancer incidence and mortality after lower endoscopy. N Engl J Med.

[12] Zauber AG, Winawer SJ, O’Brien MJ, Lansdorp-Vogelaar I, van Ballegooijen M, Hankey BF (2012). Colonoscopic polypectomy and long-term prevention of colorectal-cancer deaths. N Engl J Med.

[13] de Jong AE, Hendriks YM, Kleibeuker JH, de Boer SY, Cats A, Griffioen G (2006). Decrease in mortality in Lynch syndrome families because of surveillance. Gastroenterology.

[14] Armaroli P, Villain P, Suonio E, Almonte M, Anttila A, Atkin WS (2015). European code against cancer, 4th edition: cancer screening. Cancer Epidemiol.

[15] CBO (2008). Heriditary colorectal cancer, national guideline.

[16] Asayama N, Ikehara H, Yano H, Saito Y (2013). Endoscopic submucosal dissection of multiple flat adenomas in the radiated rectum. World J Gastrointest Endosc.

[17] Morita H, Koyama N, Tamura Y (1998). Development of flat adenoma and superficial rectal cancer after pelvic radiation. J Clin Gastroenterol.

[18] Hugen N, van Beek JJ, de Wilt JH, Nagtegaal ID (2014). Insight into mucinous colorectal carcinoma: clues from etiology. Ann Surg Oncol.

[19] van Eggermond AM, Schaapveld M, Lugtenburg PJ, Krol AD, de Boer JP, Zijlstra JM (2014). Risk of multiple primary malignancies following treatment of Hodgkin lymphoma. Blood.

[20] Aleman BM, van den Belt-Dusebout AW, Klokman WJ, Van’t Veer MB, Bartelink H, van Leeuwen FE (2003). Long-term cause-specific mortality of patients treated for Hodgkin’s disease. J Clin Oncol.

[21] Dekker N, Veer van ’t MB, Aleman BM, van Leeuwen FE, Raemaekers JM (2015). The BETER survivorship care initiative for Hodgkin lymphoma; tailored survivorship care for late effects of treatment. Ned Tijdschr Geneeskd.

[22] Stoop EM, de Haan MC, de Wijkerslooth TR, Bossuyt PM, van Ballegooijen M, Nio CY (2012). Participation and yield of colonoscopy versus non-cathartic CT colonography in population-based screening for colorectal cancer: a randomised controlled trial. Lancet Oncol.

[23] de Wijkerslooth TR, Stoop EM, Bossuyt PM, Meijer GA, van Ballegooijen M, van Roon AH (2012). Immunochemical fecal occult blood testing is equally sensitive for proximal and distal advanced neoplasia. Am J Gastroenterol.

[24] van Lier MG, Leenen CH, Wagner A, Ramsoekh D, Dubbink HJ, van den Ouweland AM (2012). Yield of routine molecular analyses in colorectal cancer patients </=70 years to detect underlying Lynch syndrome. J Pathol.

[25] Wilschut JA, Steyerberg EW, van Leerdam ME, Lansdorp-Vogelaar I, Habbema JD, van Ballegooijen M (2011). How much colonoscopy screening should be recommended to individuals with various degrees of family history of colorectal cancer?. Cancer.

[26] Loeve F, Boer R, van Oortmarssen GJ, van Ballegooijen M, Habbema JD (1999). The MISCAN-COLON simulation model for the evaluation of colorectal cancer screening. Comput Biomed Res.

[27] Loeve F, Brown ML, Boer R, van Ballegooijen M, van Oortmarssen GJ, Habbema JD (2000). Endoscopic colorectal cancer screening: a cost-saving analysis. J Natl Cancer Inst.

[28] Wilschut JA, Hol L, Dekker E, Jansen JB, Van Leerdam ME, Lansdorp-Vogelaar I (2011). Cost-effectiveness analysis of a quantitative immunochemical test for colorectal cancer screening. Gastroenterology.

[29] de Wijkerslooth TR, de Haan MC, Stoop EM, Bossuyt PM, Thomeer M, Essink-Bot ML (2012). Burden of colonoscopy compared to non-cathartic CT-colonography in a colorectal cancer screening programme: randomised controlled trial. Gut.

[30] Rubin DT, Rothe JA, Hetzel JT, Cohen RD, Hanauer SB (2007). Are dysplasia and colorectal cancer endoscopically visible in patients with ulcerative colitis?. Gastrointest Endosc.

[31] Sawhney MS, Farrar WD, Gudiseva S, Nelson DB, Lederle FA, Rector TS (2006). Microsatellite instability in interval colon cancers. Gastroenterology.

[32] Youn P, Li H, Milano MT, Stovall M, Constine LS, Travis LB (2013). Long-term survival among Hodgkin’s lymphoma patients with gastrointestinal cancer: a population-based study. Ann Oncol.

[33] Landier W, Bhatia S, Eshelman DA, Forte KJ, Sweeney T, Hester AL (2004). Development of risk-based guidelines for pediatric cancer survivors: the Children’s oncology group long-term follow-up guidelines from the children’s oncology group late effects committee and nursing discipline. J Clin Oncol.

[34] Daniel CL, Kohler CL, Stratton KL, Oeffinger KC, Leisenring WM, Waterbor JW (2015). Predictors of colorectal cancer surveillance among survivors of childhood cancer treated with radiation: a report from the childhood cancer survivor study. Cancer.

